# Retention on antiretroviral therapy in person with HIV and viral hepatitis coinfection in Ethiopia: a retrospective cohort study

**DOI:** 10.1186/s12889-022-13025-y

**Published:** 2022-04-04

**Authors:** Eleni Seyoum, Meaza Demissie, Alemayehu Worku, Adane Mihret, Alemseged Abdissa, Yemane Berhane

**Affiliations:** 1 Institute of Public Health, University of Gondar, Gondar, Ethiopia & Department of Epidemiology and Biostatistics, Addis Continental Institute of Public Health, 10433 Addis Ababa, Ethiopia; 2 Department of Epidemiology and Biostatistics, Addis Continental Institute of Public Health, Addis Ababa, Ethiopia; 3 School of Public Health, College of Health Sciences, Addis Ababa University, Addis Ababa, Ethiopia; 4 Armauer Hansen Research Institute, Addis Ababa, Ethiopia

**Keywords:** ART, Coinfection, HBV, HCV, Monoinfection, Retention

## Abstract

**Background:**

HIV coinfection with viral hepatitis B (HBV) or viral hepatitis C (HCV) is not uncommon in Ethiopia. Although the coinfections are presumed to interfere with antiretroviral treatment (ART), this is not widely studied in Sub-Saharan African settings. This study was conducted to determine ART retention in persons coinfected with HIV + HBV or HIV + HCV.

**Methods:**

We reviewed the medical records of HIV-positive adults who initiated ART between 2011 to 2018 in four high-burden hospitals of Addis Ababa. Retention in care was the primary outcome of the study, which was compared between HIV and either HBV or HCV coinfected persons, and HIV-monoinfected persons. A parametric Gompertz regression model was used to compare retention between the coinfected and monoinfected groups.

**Results:**

A total of 132 coinfected persons and 514 HIV-monoinfected individuals who initiated ART in 2011–2018 were compared. At 12-months of follow-up, 81.06% [95% CI: 73.3–86.9%] of the coinfected and 86.96% [95% CI: 83.7–89.6%] of the monoinfected were still on ART care. Cumulative retention in the coinfected group was 68.93% [60.4–76.3%] versus 80.35% [76.6–83.5%, *p* = 0.0048] in the monoinfected group. The cumulative retention was lower (61.25, 95% CI: 49.9–71.4%) in male coinfected patients than male monoinfected patients (77.77, 95% CI: 71.8–82.7%, *p* = 0.0041). In contrast, cumulative retention was similar in females in the coinfected group (80.76, 95% CI:67.3–89.5%) versus the monoinfected group (82.29, 95% CI:77.4–86.3%, *p* = 0.792). Overall, HIV-positive with viral hepatitis coinfection were 24 and 31% less likely to still be on ART care than the monoinfected group at 12 months and overall, with sub-distribution adjusted hazard ratio (AHR) of 0.76(95% CI:0.61–0.96, *p* = 0.021) and 0.69(95% CI:0.54–0.87, *p* = 0.002) respectively.

**Conclusions:**

We observed that c*oinfected individuals are less likely to stay on ART than HIV monoinfected individuals.* The low retention in the coinfected group from this study may affect the success of survival gained in people living with HIV (PLHIV) in the long term. More concerted efforts need to be made to retain coinfected individuals at least at the level of monoinfected persons on long-term ART care. Future studies are needed to better understand the difference in retention, preferable in a prospective manner.

**Supplementary Information:**

The online version contains supplementary material available at 10.1186/s12889-022-13025-y.

## Background

In the era of free antiretroviral treatment (ART), morbidity and mortality have declined in people living with HIV in resource-limited countries [1]. However, comorbidities such as hepatitis B virus (HBV) and hepatitis C virus (HCV) have become a challenge to maintain the decline in mortality [2]. Empirical evidence has shown that people living with HIV (PLHIV) taking ART, if they are coinfected with hepatitis can have serious medical complications [3–6]. However, little is known about the influence of coinfection on ART outcomes in Africa, where both viral hepatitis and HIV infections are endemic.

Globally, HBV affected an estimated 257 million people and HCV 71 million in 2015 [7, 8]. Viral hepatitis B and C are root causes of liver cancer, leading to 1.3 million deaths every year. An estimated 2.7 million PLHIV had chronic HBV infection and 2.3 million HCV in 2017 [9, 10]. This may be due to a shared mode of transmission, as both hepatitis and HIV are known to be transmitted through blood and blood products, from mother to child, and through unsafe sex [7].

Viral hepatitis B is endemic (9.4%) in Ethiopia in the general population [11]. Systematic reviews in Ethiopia showed a 5% (95% CI: 4–7%) HIV + HBV coinfection rate and 5.5% (95% CI 3.8–7.8%) HIV + HCV coinfection rate in PLHIV in 2019 and 2016 respectively [12, 13]. Despite the high burden of viral hepatitis in PLHIV, evidence on ART treatment outcomes in coinfected HIV positives are scarce in Ethiopia. Understanding ART treatment outcomes such as retention, loss to follow-up (LTFU) and mortality in the coinfected population are necessary to prioritize services and strengthen integrated care and treatment at the facility level, which will help to sustain the gains made from ART programs in the country.

This paper aimed to compare the ART outcomes in terms of ART retention in viral hepatitis coinfected (HBV-positive or HCV-positive) and HIV-monoinfected (without HBV and HCV) individuals in Addis Ababa, Ethiopia.

## Methods

### Study design and setting

This study utilized a retrospective cohort study design. The study compared ART outcomes (retention, morality, lost to follow-up) between coinfections (HBV or HCV) and monoinfection groups. As access to viral hepatitis testing in Ethiopia is low, we conducted our study in four health facilities where there was a better availability of viral hepatitis testing.

The four study facilities are considered high-HIV-burden hospitals (three public and one private) in Addis Ababa and testing facilities were better organized compared to other health facilities.

Addis Ababa is the capital city of Ethiopia and carries one-fourth of the burden of HIV in the country. An estimated 100,000 HIV-positive persons were receiving ART at the end of 2019 in the city [14]. A free ART program was initiated by the city administration in 2005.

Comprehensive HIV care, treatment, and prevention services are provided based on the national guidelines adopted from WHO recommendations. Until 2016, ART initiation was based on CD4 cell/mL counts: < 200 CD4 cells/mL from year 2005–2010, < 350 CD4 cells/mL from 2011 to 2013, and < 500 CD4 cells/mL from 2014 to 2016 [15]. In 2016, all PLHIV became eligible for ART under a “test and treat” strategy [16].

All medical care was provided by a team of physicians, nurses, adherence counselors, and pharmacists. Adherence counseling and patient tracing for lost-to-follow-up patients were supported by expert patients (HIV positive). Tracing lost-to-follow-up patients was mostly done by telephone follow-up. Full-time data clerks and a data manager supported the clinical team in handling the recording and reporting of patient data. Patients newly on ART were monitored monthly until they became stable. Once they were stable and willing, they were given an appointment spacing where they could take their drug every three to 6 months instead of monthly. In most facilities, patients received their refill drug at the ART clinic during their monthly or quarterly visits. There was a linkage between the ART clinic and pharmacy prescription records that confirmed patients picked up their drugs.

The standard first-line treatment option for HIV + HBV coinfection was the tenofovir-based combination regimen, which acts against both HIV and HBV. The WHO recommends direct-acting-drugs (DADs) for treating chronic HCV-coinfected patients. However, DAD is very expensive, and treatment services are not available in most ART health facilities, including the study facilities of Addis Ababa. So, HCV coinfection patients in our data is lacking information on HCV treatment.

### Study population

The study population comprised PLHIV who started on ART between September 2011 and December 2018. Participants were included if they were aged 15 or older and had documented viral hepatitis B surface antigen and viral hepatitis C antibody test results, regardless of whether the result of viral hepatitis B and C was positive or negative. Accordingly, of the total 3006 eligible in the four study health facilities we were able to include 2031 in our analysis.

### Primary outcome

*Retention:*

- Retention at 12 months: individuals who were alive and still taking ART 12 months after ART initiation.
- Cumulative retention: individuals who were alive and on ART for the whole follow-up period [17].
- Alive and on ART (still on ART): patients who picked up their last eligible drug dose of the study period from an ART clinic/pharmacy. Patient drug pick-up was updated on follow-up cards and ART cohort registers monthly based on the patient’s monthly status.

### Secondary outcome

*Lost to follow-up (LTFU):* missing of three consecutive clinic visits (drug not picked up for 3 months or 90 days). This is captured from the patient cohort follow-up card and ART cohort register which were updated based on monthly patient status.

- LTFU at 12 months: individuals who had missed their clinic visits for three consecutive scheduled visits (90 days) at 12 months after ART initiation. Cumulative LTFU: individuals who missed their drug pick-up for three consecutive scheduled visits (90 days) at any time in follow-up period.

*Death*: Confirmed hospital deaths were reported in the ART register and patient’s follow-up cards.

- Death at 12 months: individuals who were reported dead at 12 months after ART initiation
- Cumulative death: individuals who were reported dead after ART initiation at any time in follow-up period.

### Time of coinfection

- Knowing the timing of viral hepatitis HBV and HCV coinfection is essential in measuring retention on antiretroviral therapy in PLHIV. Approximately 90% of chronic HBV in the current adult population of African and Asia started with childhood infection, most likely before HIV infection (that is, they were born before HBV vaccination began). On the other hand, in the absence of an HCV RNA diagnosis, it is not possible to ascertain whether an HIV/HCV coinfection is recent. Hence, in this study, we took the date of a new HIV diagnosis as a proxy date of coinfection for either HBV or HCV.

### Covariates

Coinfection: the presence of at least one viral hepatitis (HIV + HBV or HIV + HCV).

Monoinfection: without hepatitis B and without hepatitis C viruses (HIV only).

The following covariates were also considered based on our previous ART knowledge: age, sex, education, marital status, WHO clinical staging, baseline CD4 cell/mL count, tuberculosis status at baseline.

### Data collection and procedure

A standard data abstraction form was prepared to extract individual-level data that were pertinent to meet objectives of this paper. The form had four sections a) sociodemographic information; b) HIV care information at enrollment including; date of confirmed HIV positivity, ART initiation date, and date of each outcome; c) HIV care at baseline and follow-up, including CD4 cells/mL at baseline, at 6 months, and 12 months; WHO clinical staging; functional status; hemoglobin; liver functional test; viral hepatitis status (B and C); tuberculosis status; and drug patterns; and d) outcome variables, including survival and follow-up, death, lost to follow-up, stopping treatment, and transfer out.

Data abstractors were trained for 3 days by the principal investigator. The first 2 days of the training were spent on the data abstraction tools and how to abstract data ethically. On the third day of the training, the data abstraction tool was pretested in one of the hospitals. Data abstraction was done using a preloaded data form on a tablet. The ART register was used to select age 15+, ART initiation date, and unique ART number (that linked to the patient’s folder).

Each patient’s folder was accessed to abstract laboratory information, including CD4 cell/mL count, viral load, ALT, AST, platelets, hemoglobin, WHO stage, viral hepatitis B and C, and monthly follow-up visit status. The nurses abstracted the clinical and laboratory data, and the data managers were responsible for direct data entry into the tablet. Extracted data were uploaded daily to the REDCAP server after being checked by a trained supervisor, and the study team provided regular supervision at the study site.

### Sample size and sampling procedure

A power calculation was done to ensure whether the available records would be enough to address the main objective of this study. The coinfected group was anticipated to be much smaller than the monoinfected group, as the criterion for starting ART was mainly HIV positivity. Thus, a ratio of 1 coinfected to 4 monoinfected was used. The sample size was estimated for the two outcomes (retention and mortality), and the largest estimated sample size (mortality) is presented. Mortality in HIV + HBV (hazard rate of 1.4) and HIV + HCV (hazard ratio of 1.4) was taken from a previous study [18]. A hazard ratio of 1.4, the coinfected hazard rate (2.2) versus the monoinfected hazard rate (1.6), a significance level of α = 0.05, a 0.25 proportion of (coinfected to monoinfected), and 80% power was applied to the STATA11 Exponential test, hazard with the commands stpower exponential 1.6, hratio 1.4, nratio0.25, and loghazard applied. Based on the input parameter, an estimated sample size of 410 (82 exposed and 328 nonexposed) was calculated to carry out this study. We expected different proportions of study participants with coinfection and monoinfection. From the previous study, the proportion of coinfection is very low. Hence, we increased the total sample size but kept a smaller sample in the coinfection group and 4 times that sample in the monoinfected group to obtain the same statistical power we would have reached if we had assumed a 1:1 ratio. Going beyond a 1:4 ratio would not increase the efficiency of the study, and there would little extra gain in the power from recruiting more than 4 coinfected patients for every monoinfected patient. To increase the precision and power of the study, all coinfected (132) and randomly selected (514, 1:4 ratio) monoinfected persons on ART were included from the cohort of September 2011 to December 2018.

### Data analysis

The background and clinical characteristics were summarized as the median and IQR for nonnormally distributed numeric variables, and as proportions and frequencies for categorical variables. Baseline clinical variables were compared using the chi-square test for categorical variables and the Kruskal-Wallis or Wilcoxon test for nonnormally distributed continuous variables, as appropriate. Proportions were calculated for ART retention, loss to follow-up, and mortality and are presented with their respective *p*-values. To calculate retention, we used the last day of the study as a right censor, and events that occurred were death and loss to follow-up together*.* Overall, 15% of the patients were tested but had no HBV/HCV test results mentioned and excluded from the analysis. We checked potential bias by comparing demographic and clinical characteristics between patients who were tested for viral hepatitis B and C versus those without document test result and we did not find any important significant differences in demographic and clinical data (Supplement Table 1)*.* Data completeness for the primary outcome and covariates were compared between the two groups with the chi-square test *(*Supplement Table 2). To adjust for potential confounders, parametric regression modeling was performed on the primary and secondary outcomes. Evidence shows that parametric regression models have often been more reliable and less biased than Cox regression models [19]. Proportional Hazard global test was used to check for proportionality with command PH (estat phtest) for retention at 12 months and obtained Global test of Prob>chi2 = 0.9493. Model distributions were compared using Akaikie’s information criterian (AIC) and the Log likelihood test. The Gompertz parametric regression model with the lowest AIC and highest Log likelihood value was selected in the final analysis (Supplement Table 3). Statistical analysis was performed using STATA14 software. A hazard of cumulative retention (stratified by sex) was presented by the ART follow-up period. The hazard ratio was adjusted for sex, age at ART initiation (15–29, 30–44, 45+), education (no education, primary, secondary, and tertiary), CD4 cells/mL at the start of ART (< 200, 201–350, > 350 cells/μL), tuberculosis (yes or no), WHO stage (I&II versus III&IV). The type of facility (public versus private) was used as a group variable to account for a random effect, in the shared frailty model. In addition, death was considered as a competing risk of an event in the analysis as it might preclude the occurrence of the primary event of interest (retention). We used the Sub-distribution model to calculate the hazard ratio and compared it without considering competing for risk as an event (Supplement Tables 4a & 4b). Sensitivity analysis of retention was carried out by excluding all HIV-TB coinfections from both coinfected and monoinfected groups and excluding HCV (Supplement Table 5a & 5b) but did not show any significant influence on the main study finding. The study was approved by the Institutional Review Board of the University of Gondar, and we affirm that all methods were performed in accordance with the relevant guidelines.

## Result

### Background characteristics of study participants

**Fig. 1:**
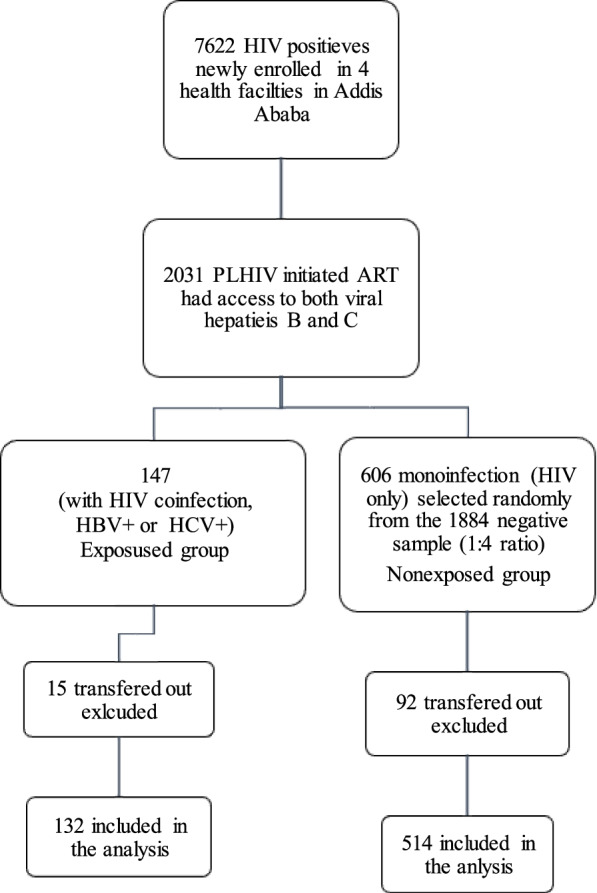
Summary of eligible study participants and conditions for exclusion, from the cohort of 2011 to 2018 in Addis Ababa

We compared 132 coinfected (HIV and hepatitis) and 514 monoinfected (only HIV-positive) who started on ART during the study period (Fig. 1). The median follow-up time was 3.25 years (IQR, 1.8–5.3) for monoinfected and 3.23 years (IQR, 1.6–6.0) for coinfected group. The coinfected group had a significantly higher median age (39 years, IQR 34–45), than the monoinfected group (36 years, IQR 30–43, *p* = 0.002). The proportion of males was significantly higher (60.61%) in the coinfected group than in the monoinfected group (43.86%) (Table 1).

**Table 1:** Baseline demographic characteristics and health factors of participants who initiated ART by coinfection status in four hospitals in Addis Ababa, Ethiopia (September 2011 to December 2018), *n* = 646

Characteristics	n (%) of participants	
	HIV Monoinfection (*n* = 514)	HIV Coinfection (*n* = 132)
Sex (*n* = 645) ^a^		
Female	288(56.14)	52 (39.39)
Male	225(43.86)	80 (60.61)
Age (years), (n = 646)		
Median (IQR)^a^	36(30–43)	39(34–45)
15–29	116(22.57)	15(11.36)
30–44	284(55.25)	82(62.52)
> =45	114(22.18)	35(26.52)
Marital status, (*n* = 623)		
Never married	108(21.69)	19(15.20)
Married	249(50.00)	74(59.20)
Divorced/separated	92(18.47)	19(15.20)
Widow/er	49(9.84)	13(10.40)
Education (n = 623)		
No education	68(13.74)	22(17.32)
Primary	168(33.94)	35(27.56)
Secondary	166(33.54)	49(38.58)
Tertiary	93(18.78)	21(16.54)
Median follow-up-time (in years)	3.25(1.8–5.3)	3.23(1.6–6.0)
ART regimen at start of ART (*n* = 634)		
Tenofovir based	465(92.08)	117(90.70)
Non-Tenofovir based	40(7.92)	12 (9.30)

HIV-Monoinfection: negative for both hepatitis B virus and hepatitis C viruses

HIV-Coinfection: hepatitis B virus or hepatitis C virus positive

*IQR* interquartile range; *WHO* World Health Organization, *ALT* alanine transaminase, *AST* aspartate aminotransferase

^a^Indicates significant difference between the HIV-coinfected and HIV-monoinfected groups with a *p-value* < 0.05

More than half of the coinfected (63, 52.07%) and monoinfected (263, 54.68%) initiated ART with CD4 cells/mL counts below 200. Similarly, more than half of the coinfected group (73, 56.15%) and monoinfected group (261, 51.28%) initiated ART at WHO stage III or IV. At baseline monoinfected individuals more often had TB (168,32.71%) than coinfected patients (17, 13.71%, *p* = 0.001). A higher median AST (36, IQR 26.0–53.5) and ALT (28, IQR 18–44) were found in the coinfected group than the monoinfected group (30.5, IQR 22–45, *p* = 0.0032; and 23, IQR 16–38, *p* = 0.0406, respectively) (Table 2).

**Table 2:** Baseline clinical and health factors of patients initiating ART by coinfection in four high-burden hospitals in Addis Ababa, Ethiopia (September 2011 to December 2018), n = 646

Characteristics	n (%) of participants	
	HIV Monoinfection	HIV Coinfection
CD4 cell count/mm^3^ (*n* = 602)		
Median (IQR)	181(97–310)	183(87–318)
< 200	263(54.68)	63(52.07)
200–350	129(26.82)	34(28.10)
> 350	89(18.50)	24(19.83)
WHO staging (*n* = 639)		
I & II	248(48.72)	57(43.85)
III & IV	261(51.28)	73(56.15)
Functional status (*n* = 635)		
Bedridden	81(15.98)	16(12.50)
Ambulatory	26(5.12)	8(6.25)
Working	400(78.90)	104(81.25)
^b^Tuberculosis (TB) presence at baseline		
Yes	168(32.71)	17(13.71)
No	346(67.32)	107(86.29)
Opportunistic infection (OI) at baseline, *n* = 514		
Yes	201(39.11)	74(56.06)
No	313(60.89)	58(43.94)
Median hemoglobin (IQR) (*n* = 474)	13(11.4–14.5)	13.9(12.1–15.1)
^b^ Median AST (IQR) (*n* = 549)	30.5(22–45)	36(26–53.5)
^b^ Medina ALT (IQR) (*n* = 508)	23(16–38)	28(18–44)
^b^ Median Platelet (IQR) (*n* = 260)	242(198–315)	185(151–218)

HIV-Monoinfection: negative for both hepatitis B virus and hepatitis C viruses; HIV-Coinfection: hepatitis B virus or hepatitis C virus positive; *IQR* interquartile range, *WHO* World Health Organization, *ALT* alanine transaminase, *AST* aspartate aminotransferase; ^b^ Indicates significant difference between the HIV-coinfected and HIV-monoinfected groups with a *p-value* < 0.05

### Retention in care at 12 months

The proportion of retention in care at the 12-month follow-up in coinfected group was 81.06% [95% CI:73.3–86.9%] versus 86.96% [95% CI: 83.7–89.6%] in monoinfected group. Retention at 12 months was lower (77.5, 95% CI: 66.8–85.4%) in the male coinfected subgroup than in the male monoinfected subgroup, (86.66%, [95% CI: 81.5–90.5%, *p* = 0.0513). On the other hand, retention at 12 months in females in the coinfected group was, similar (86.53, 95% CI; 73.8–93.6%) to that in the monoinfected group, (87.15, 95% CI: 82.7–90.5% *p*-value = 0.903) (Table 3).

**Table 3:** Proportion of retention and attrition after ART initiation in the follow-up period by coinfection status and gender, in Addis Ababa, Ethiopia, (September 2011 to December 2018)

Outcome	Coinfection, (n = 132)			Monoinfection, (*n* = 514)		
	Male	Female	Total	Male	Female	Total
n (%), 95%: CI						
***Retention***						
at 12 months	62(77.50) [66.8–85.4]	45(86.53) [73.8–93.6]	107(81.06) [73.3–86.9]	195(86.66) [81.5–90.5]	251(87.15) [82.7–90.5]	446(86.96) [83.7–89.6]
Cumulative retention	80(61.25) [49.9–71.4]	52(80.76) [67.3–89.5]	132(68.93) [60.4–76.3]	225(77.77) [71.8–82.7]	289(82.29) [77.4–86.3]	514(80.35) [76.6–83.5]
***LTFU***						
LTFU at 12 months	17.5 [10.5–27.6]	7.69 [2.8–19.3]	13.63 [8.7–20.7]	10.66 [7.2–15.4]	9.72 [6.7–13.7]	10.11 [7.7–13.0]
Cumulative LTFU	31.25^c^ [20.8–41.6]	13.46^c^ [3.8–23.0]	24.24^d^ [16.8–31.6]	19.55 [14.3–24.7]	14.58 [10.4–18.6]	16.73^d^ [13.4–19.9]
***Death***						
Death at 12 months	5.0 [1.8–12.8]	5.76 [1.8–16.97]	5.3^e^ [2.5–10.8]	2.66 [1.19–5.8]	3.12 [1.-6–5.9]	2.91^e^ [1.76–4.7]
A cumulative death	7.5 [3.3–15.9]	5.76 [1.8–16.9]	6.81 [3.5–12.6]	2.66 [1.1–5.8]	3.12 [1.6–5.9]	2.91 [1.7–4.7]

HIV-Monoinfection: negative for both hepatitis B virus and hepatitis C viruses; HIV-Coinfection: hepatitis B virus or hepatitis C virus positive; *LTFU* loss to follow-up ^c^ Compared LTFU between male and female in co-infected group and there is a significant difference with a *p-value <* 0.0203 ^d^ compared LTFU in coinfected versus monoinfected and there is a significant difference with a *p*-value (0.0318). ^e^ Compared death in coinfected versus monoinfected and there is a significant statistical association

### Cumulative retention

**Fig. 2:**
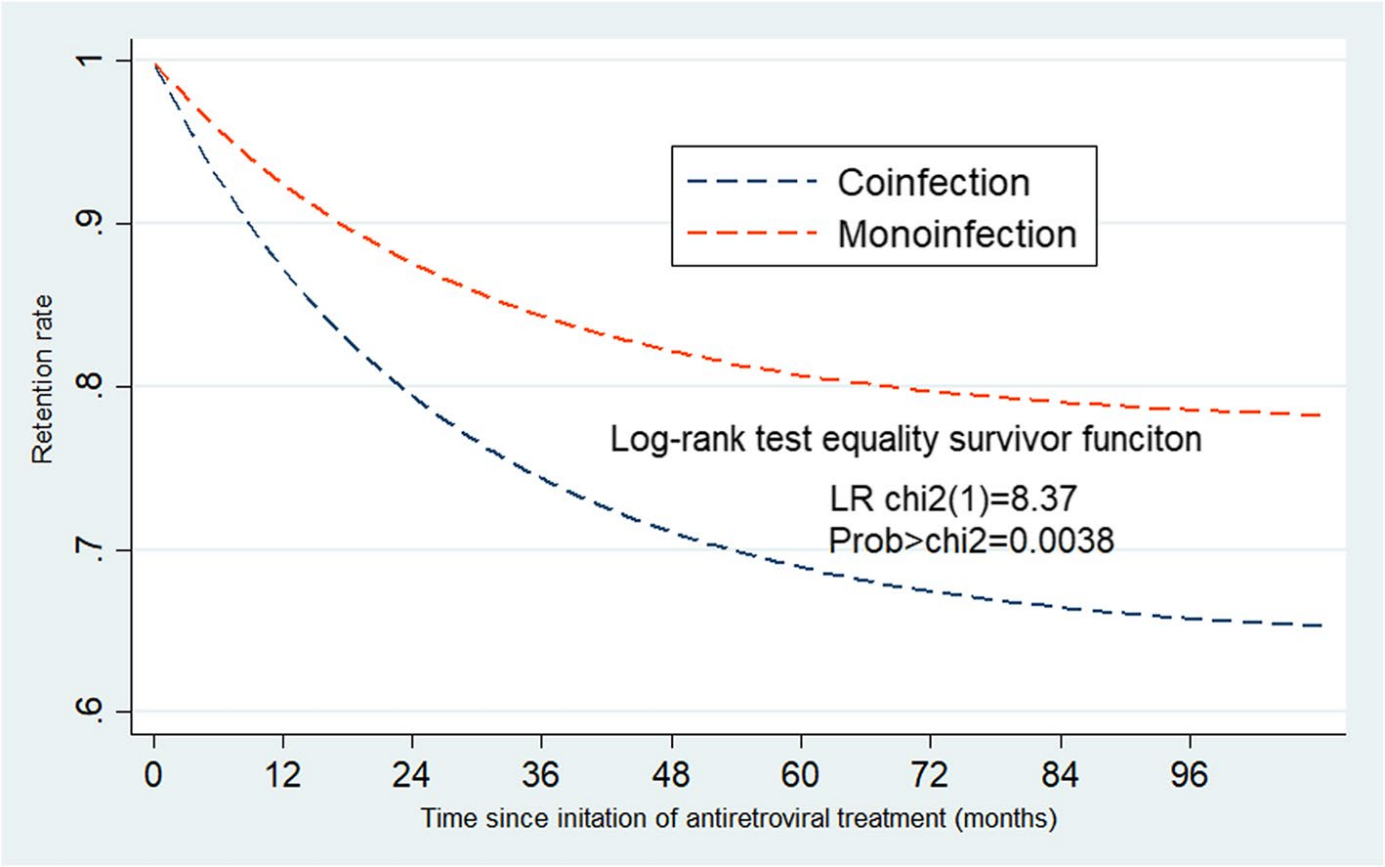
Retention among adults receiving antiretroviral treatment by follow-up time in Addis Ababa

The cumulative retention in the coinfected group was 68.93% [60.4–76.3%] versus 80.35 [76.6–83.5%, *p* = 0.0048] in the monoinfected group. The cumulative retention was lower (61.25, 95% CI: 49.9–71.4%) in the male coinfected subgroup than in the male monoinfected subgroup (77.77, 95% CI: 71.8–82.7%, p-value = 0.0041). In contrast, cumulative retention was similar in females in the coinfected; (80.76, 95% CI:67.3–89.5%) and the monoinfected group (82.29, 95% CI, 77.4–86.3, *p* = 0.792) (Table 3). Figure 2 illustrates the retention by follow up time. The Log-rank test of equality of survivor function shows a significant difference in retention between coinfection and monoinfection individuals at each follow-up time with (*p* = 0.0038, and corresponding *x*^2^ = 8.37).

### Loss to follow-up and death

Over the entire follow-up time, a total of 141 participants stopped ART care (121 were lost-to-follow-up and 24 died). The proportion of LTFU at the 12-month after ART started in coinfected group was 13.63% [95% CI:8.7–20.7%] versus 10.11% [95% CI: 7.7–13.0%] in monoinfected group. LTFU at 12 months was higher (17.5, 95% CI: 10.5–27.6%) in the male coinfected subgroup than in the male monoinfected subgroup, (10.66% [95% CI: 7.2–15.4%). Coinfected individuals had a higher cumulative LTFU, 24.24% [16.8–31.6%] than the monoinfected group, 16.73% [13.4–19.9%, *p* = 0.0465]. The cumulative LTFU was higher (31.25, 95% CI: 20.8–41.6%) in the male coinfected subgroup than in the male monoinfected subgroup (19.55, 95% CI: 14.3–24.7%, *p*-value = 0.0321). Similarly, the death rate at 12 months in the coinfected group was 5.3% (95% CI: 2.5–10.8%), higher than that in the monoinfected group, 2.91% (95% CI:1.76–4.79%, *p* = 0.0346). The cumulative death rate was higher (6.81, 95% CI: 3.5–12.6%) in the conoinfected group than that in monoinfected group (2.91, 95% CI:1.76–4.79) (Table 3).

**Fig. 3:**
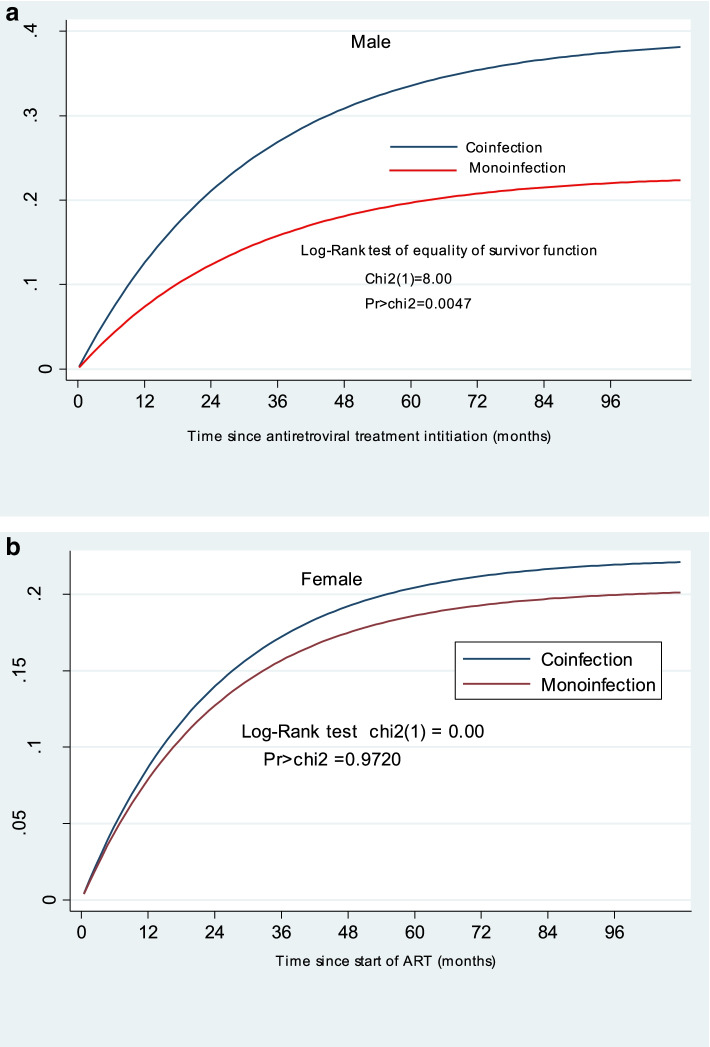
**a** & **b** Cumulative attrition rate in males and females aged 15+ since start of ART by coinfection status

The cumulative attrition (death and LTFU together) in the male subgroup of the coinfected group was higher than the cumulative attrition rate in the male subgroup of the monoinfected group with a Log-rank test equality of survival function, *p* = 0.0047 and *x*^2^ = 8.00 (Fig. 3a). In contrast, attrition was similar between coinfected and monoinfected females, *p* = 0.972 and *x*^2^ = 0.00(Fig. 3b).

### Adjusted retention and attrition hazard ratios

The adjusted hazard ratio showed that PLHIV who had coinfection with hepatitis were 24% less likely to stay on ART care than the monoinfected group at 12 months, with an adjusted hazard ratio of 0.76(0.61–0.96, *p* = 0.021), and 31% stopped ART at some time in the whole follow-up period (cumulative), with sub-distribution adjusted hazard ratio of 0.69 (0.54–0.87, *p* = 0.002) in the coinfected group. LTFU at 12 months was 63% higher in coinfected group, with an adjusted hazard ratio of 1.63 (95% CI: 0.95–2.82, *p* = 0.075); similarly, the cumulative loss during the whole follow-up period was 1.67 (95% CI:1.07–2.63, *p* = 0.025). On the other hand, death at 12 months in the coinfected group was 1.90-fold (95% CI; 0.71–5.09, *p* = 0.197) than that in the monoinfected group. The cumulative death rate in the coinfected group was 2.49 times that in the coinfected group (adjusted hazard ratio 2.49 (1.02–6.10) (Table 4).

**Table 4:** Adjusted and unadjusted hazard ratio of outcomes in coinfection with use of parametric Gompertz regression model, in Addis Ababa, Ethiopia, (September 2011 to December 2018), *n* = 568

Outcome	Crude Hazard Ratio			Adjusted Hazard Ratio ^b^		
	Haz. Ratio [95% CI]	Standard Error	*p-value*	Haz. Ratio [95% CI]	Standard Error	*p-value*
***Retention***						
at 12 months	0.80(0.65–0.99)	0.086	0.044	0.76(0.61–0.96)	0.088	0.021
Cumulative retention	0.73(0.58–0.92)	0.085	0.008	0.69(0.54–0.89)	0.090	0.004
^a^SRH Cumulative retention	0.68(0.55–0.85)	0.076	0.001	0.69(0.54–0.87)	0.083	0.002
***Attrition (overall)***						
LTFU at 12 months	1.51(0.95–2.40)	0.3556	0.075	1.63 (0.95–2.82)	0.4546	0.075
Cumulative lost	1.47(0.98–2.20)	0.3049	0.062	1.67(1.07–2.63)	0.3857	0.025
Death at 12 months	1.89(0.77–4.65)	0.8649	0.162	1.90(0.71–5.09)	0.9565	0.197
Cumulative death	2.44(1.06–5.57)	1.0288	0.034	2.49(1.02–6.10)	1.140	0.045

^a^Sub-distribution hazard ratio model is used considering death as a competing risk of event in the analysis for cumulative retention. However, at 12 months no competing risk was reported

^b^The model was adjusted for age, sex, marital status, education, baseline cd4 count, facility type (by shared frailty model); LTFU: Loss to follow-up

## Discussion

In this study, we observed low retention, high loss to follow-up, and death in the coinfected group compared with the monoinfected group. The rate of retention on ART was significantly lower in males than females. Retaining patients in care is crucial to achieve the desired outcome of ART and to meet the global target of 90% of ART retention [20]. Evidence on retention, mortality, and loss to follow-up in coinfected groups is not widely available in Ethiopia. So direct comparison with other studies may not be possible. However, comparing our findings with the overall retention rate may provide some clues about both monoinfected and coinfected groups. The 12 month retention in this study for the monoinfected group demonstrated similar findings as the retrospective cohort studies in Addis Ababa in 2015 (86%) and 2016 (79%) [21, 22]. Our observed retention in the coinfected group was much lower than the national target (90%) 12 month retention by 2020 for all HIV positive individuals, including coinfected individuals [23], while for the monoinfected group, the retention was on track.

The higher attrition (LTFU and death) rate in the coinfected group could be the result of many factors. The absence of coinfected integrated prevention, care, and treatment services for viral hepatitis may lead coinfected individuals to drop out of treatment in search of viral hepatitis services which are limited to a few hospitals. The high attrition rate in the coinfected group found in this study may affect the long-term survival gained from ART in PLHIV and may affect the progress in reaching the global targets. The two previous studies in Addis Ababa showed an overall attrition rate of 21.92% (15.17% loss to follow-up and 6.5% death) [21, 22]. These numbers are similar to those of the monoinfected group in this study but significantly lower than those found in the coinfected group. A systematic review in Ethiopia showed an overall attrition of 21% in HIV-positive people enrolled for ART care, which is also comparable with the monoinfected cumulative attrition in this study even after excluding those transferred out from our analysis; the other study considered transferred-out patients to be retained on ART [24]. Therefore, direct comparison with this study may not be reasonable.

Our study found twofold higher mortality in the coinfected group. A comparable result was documented in a South African retrospective cohort study with a sample size of 816 [25]. Cambodia’s large study with a sample size of 3089 in both HBV and HCV showed 1.6 higher mortality for HBV coinfection and the risk of mortality was a 3-fold increase in HCV coinfection [26]. A higher risk of mortality was also reported in an Asian cohort coinfected group [4].

Even in countries with a much better health care system death in coinfected groups is high, a 10 year trend analysis done in France shows a result which is consistent with our finding; increased death in coinfected HIV patients than monoinfected [27].

Our findings showed a significantly lower rate of retention in males than in females in the coinfected group. In contrast, retention was similar between males and females in the monoinfected group. The lower retention rate and the high loss to follow-up in males with viral hepatitis B or C can be explained by the fact that female HBV carriers have lower viral loads than male carriers [28]. HBV-associated complications, including hepatocellular carcinoma (HCC), develop more frequently in men than in women [29]. Studies have also shown that elevated testosterone levels are associated with an increased risk of hepatocellular carcinoma [30].

The significantly increased mean AST and ALT may lead to intolerance of ART drugs and dropout from care [31–33]. Similar findings have also been reported in China [34]. The elevated liver enzymes of AST and ALT in the coinfected group may provide a clue about liver-related diseases in the coinfected groups, which may call for further investigation in the Ethiopian ART cohort. HIV coexists with viral hepatitis in a fairly large proportion of Ethiopians.

Some of the limitations of this study include the inability to control for other potential confounders, such as stage of liver disease, cause of death, adherence to a TDF-based drug regimen, and alcohol use. Viral load was selected as a covariant, however, more than 65% of the patients had no viral load test at 6 or 12 months. Due to the high level of missing values viral load was omitted from the analysis. Information on mode of HIV transmission was not available in this study. However, in Ethiopia mode of HIV transmission is primarily via heterosexual contact among adults.

The absence of HCV RNA diagnosis can increase the false positive rate of chronic HCV patients, particularly in the non-HIV population. In contrast, HBsAg underestimates chronic HBV cases because it misses occult HBV that can only be detected by HBV DNA. Hence**,** the reliability of the diagnostic tools remains the limitation of the study as well.

The loss-to-follow-up criteria of more than 3 months in this study are likely to underestimate mortality and overestimate loss to follow-up compared to other studies found [35, 36]. As the underestimation of death and overestimation of loss to follow-up affect both groups, this should not alter our results. However, interpretation of the death and loss to follow-up in this study must consider these study limitations. Another limitation of a study worth mentioning is the selection of study sites and study participants based on the availability of viral hepatitis test. Although that remains a threat to selection bias comparison of those excluded with those included in the analysis by age and sex showed no statistically significant difference.

## Conclusion

We observed that c*oinfected individuals are less likely stay on ART than HIV monoinfected individuals.* The low retention in the coinfected group from this study may affect the success of survival gained in people living with HIV (PLHIV) in the long term. More concerted efforts need to be made to retain coinfected individuals at least at the level of monoinfected persons on long-term ART care. Future studies are needed to better understand the difference in retention, preferable in a prospective manner.

## Supplementary Information

**Additional file 1.**

**Additional file 2.**

**Additional file 3.**

**Additional file 4.**

**Additional file 5.**

## Data Availability

The datasets used and/or analyzed during the current study are available from the corresponding author on reasonable request.

## Abbreviations

ALT: Alanine aminotransferase
ART: Antiretroviral treatment
AST: Aspartate aminotransferase
HBV: Hepatitis B virus
HCV: Hepatitis C virus
HBsAg: Hepatitis B surface antigen
HMIS: Health Management Information System
IQR: Inter-quartile range
PLHIV: People Living with HIV
WHO: World Health Organization
