# Supplementary material for: Retention on antiretroviral therapy in person with HIV and viral hepatitis coinfection in Ethiopia: a retrospective cohort study
Source: BMC Public Health. 2022 Apr 4;22:644. doi: 10.1186/s12889-022-13025-y (PMC8978407; doi:10.1186/s12889-022-13025-y)
Supplement: Supplementary file 1 — Additional file 1. [file 12889_2022_13025_MOESM1_ESM.docx]

Supplement Table 1: Percent distribution of background and clinical characteristics of ART patients who were tested for HBV/HCV and those who were not tested in Addis Ababa, n=2382

|  | Tested for HBV/HCV  % | Not tested for HBV/HCV  % | P-value |
| --- | --- | --- | --- |
|  | N=2031 | N=351 |  |
| Median Age (IQR) (years) | 36(30-43) | 35(29-42) | 0.1786 |
| Age group |  |  |  |
| 15-29 | 22.1(20.3-23.9) | 27.1(22.7-32.0) |  |
| 30-44 | 54.9(52.8-57.1) | 52.2(47.0-57.4) |  |
| 45-59 | 19.6(17.8-21.3) | 17.7(14.0-22.0) |  |
| 60+ | 3.4(2.6-4.2) | 2.8(1.5-5.2) |  |
| Sex |  |  | 0.8491 |
| Male | 42.9 (40.7-45.0) | 42.2(37.1-47.5) |  |
| Female | 57.1(54.9-59.2) | 57.7(52.4-62.8) |  |
| Marital status |  |  | 0.9557 |
| Never married | 23.0(21.1-24.9) | 33.6(28.5-39.2) |  |
| Married | 54(51.4-56.3) | 49.6(44.0-55.3) |  |
| Separated/Divorced | 3.9(3.1-4.9) | 2(0.8-4.3) |  |
| Widowed | 16.4(14.7-18.2) | 14.6(11.0-19.1) |  |
| Education |  |  | 0.2308 |
| No Education | 15.4 (13.8-17.1) | 11.9(8.8-15.9) |  |
| Primary | 32.8(30.8-34.9) | 27.3(22.7-32.4 |  |
| Secondary | 33.2(31.1-35.3) | 42(37.0-47.7 |  |
| Tertiary | 18.4(16.8-20.2) | 18.4(14.5-23.0) |  |
| CD4 category |  |  | 0.2999 |
| <200 cells/mL | 49.8(47.6-52.1) | 51.6(46.2-57.0) |  |
| 201-350 cells/mL | 26.2(24.3-28.3) | 31.3(26.5-36.5) |  |
| 351-500 | 12.6(11.1-14.1) | 10.3(7.4-14.1) |  |
| >500 | 11.3(9.8-12.7) | 6.6(4.4-9.9) |  |
| WHO clinical stages |  |  | 0.0882 |
| Stage 1 | 41.0 (38.8-43.1) | 34.6(29.8-39.8) |  |
| Stage 2 | 19.2 (17.4-20.9) | 23.4(19.3-28.3) |  |
| Stage 3 | 24.5(22.6-26.4) | 21.7(17.7-264) |  |
| Stage 4 | 15.3(13.9-16.9) | 20.0(16.1-24.6) |  |
