# Supplementary material for: Retention on antiretroviral therapy in person with HIV and viral hepatitis coinfection in Ethiopia: a retrospective cohort study
Source: BMC Public Health. 2022 Apr 4;22:644. doi: 10.1186/s12889-022-13025-y (PMC8978407; doi:10.1186/s12889-022-13025-y)
Supplement: Supplementary file 2 — Additional file 2. [file 12889_2022_13025_MOESM2_ESM.docx]

**Supplement Table 2.** Completeness of variables (covariant and outcome variables) included in the analysis, Addis Ababa, Ethiopia, (September 2011 to December 2018)

| Variables | Total  n=646, % | | Monoinfected  n=514, % | | Coinfected  n=132, % | |
| --- | --- | --- | --- | --- | --- | --- |
| Age | 646 | 100.00 | 514 | 100.00 | 132 | 100.00 |
| Sex | 645 | 99.84 | 513 | 99.80 | 132 | 100.00 |
| Marital status | 623 | 96.43 | 498 | 96.88 | 125 | 94.69 |
| Education | 622 | 96.28 | 495 | 96.30 | 127 | 96.21 |
| Baseline CD4 | 602 | 93.18 | 481 | 93.57 | 121 | 91.66 |
| WHO staging | 630 | 97.52 | 509 | 99.02 | 130 | 98.48 |
| Functional status | 635 | 98.29 | 507 | 98.63 | 128 | 96.96 |
| ^h^ Outcome variables | 646 | 100.00 | 514 | 100.00 | 132 | 100.00 |
| ^i^ Follow-up-time | 646 | 100.00 | 514 | 100.00 | 132 | 100.00 |
| ^j^ Covariant & outcomes | 633 | 97.94 | 505 | 98.24 | 129 | 97.56 |
| ^k^ Other variables | | | | | | |
| ART regimen | 634 | 98.14 | 505 | 98.24 | 129 | 97.72 |
| Hemoglobin | 592 | 91.64 | 474 | 92.22 | 118 | 89.39 |
| AST | 549 | 83.68 | 441 | 85.79 | 108 | 83.07 |
| ALT | 508 | 78.63 | 410 | 79.76 | 98 | 74.24 |
| Platelet | 260 | 40.24 | 213 | 41.43 | 47 | 35.60 |
| Average of all data | 588.42 | 91.88 | 473.46 | 91.54 | 118.5 | 89.85 |

^h^ Outcome variables include living and under follow-up, lost to follow-up and dead

^i^ Follow up time: Date of antiretroviral initiation, date of either event (death or loss) or date of study end

^j^ Average of covariate and outcome variable data completeness

^k^ Variables not included as covariates in the analysis such as: ALT=alanine transaminase, AST= aspartate aminotransferase; ART regimen, Hemoglobin, Platelet

WHO: World Health Organization
