# Supplementary material for: Retention on antiretroviral therapy in person with HIV and viral hepatitis coinfection in Ethiopia: a retrospective cohort study
Source: BMC Public Health. 2022 Apr 4;22:644. doi: 10.1186/s12889-022-13025-y (PMC8978407; doi:10.1186/s12889-022-13025-y)
Supplement: Supplementary file 3 — Additional file 3. [file 12889_2022_13025_MOESM3_ESM.docx]

**Supplement Table 3.** Comparison of Akaike’s information criterion between the Cox proportional hazard model and parametric model

| Model | Log likelihood | AIC | Kappa value |
| --- | --- | --- | --- |
| Cox | -603.65 | 1227.31 |  |
| Exponential | -424.41 | 870.824 |  |
| Weibull | -405.46 | 834.926 | ^l^Kappa=0.313, Kappa test, [kappa]_cons =1, chi2(1) = 6.71, Prob > chi2 =0.0096 |
| Gompertz | -411.76 | 847.52 |  |
| Lognormal | -401.58 | 827.16 |  |
| Loglogistic | -404.82 | 833.64 |  |
| Generalized Gamma | -399.92 | 825.84 |  |

**AIC: Akaike’s information criterion**

Model comparison was presented based on AIC and Log-likelihood value. Lower AIC and higher Log-Likelihood is used to select the best model. However, for Weibull distribution to be legitimate parametric test (Kappa-value >0.5) is required in addition to the AIC value and Log-likelihood value. The legitimacy of the Weibull distribution is tested with the STATA command “ test [kappa]_cons=1, (1) [kappa]_cons = 1, chi2 (1) = 6.71, Prob > chi2 = 0.0096. ^l^Kappa value of 0.313 is significantly lower than 1, indicating that Weibull is not legitimate for this distribution**.**
