# Supplementary material for: Retention on antiretroviral therapy in person with HIV and viral hepatitis coinfection in Ethiopia: a retrospective cohort study
Source: BMC Public Health. 2022 Apr 4;22:644. doi: 10.1186/s12889-022-13025-y (PMC8978407; doi:10.1186/s12889-022-13025-y)
Supplement: Supplementary file 4 — Additional file 4. [file 12889_2022_13025_MOESM4_ESM.docx]

Supplement Table 4a. Adjusted cumulative retention hazard ratio of outcomes in coinfected patient; in Addis Ababa, Ethiopia, (September 2011 to December 2018), n=568

_t Haz. Ratio Std. Err. P>z [95% Conf. Interval]

Hepatitis .6995826 .0872284 0.004 .5479057 .8932483

sex .9071335 .0960616 0.357 .7371105 1.116374

age 1.240538 .096517 0.006 1.065086 1.444893

baselinecd4 1.266043 .0845629 0.000 1.110693 1.443121

education 1.128607 .0579422 0.018 1.020569 1.248082

Mstatus .9322737 .0560736 0.244 .8286023 1.048916

_cons . 0021887 .0006627 0.000 .0012091 .0039621

/gamma .0325741 .0017269 0.000 .0291894 .0359588

/ln_the -3.017106 1.261734 0.017 -5.49006 -.5441522

theta .0489427 . 0617526 .0041276 .5803336

Supplementary Table 4b: Sub-distribution hazard ratios for Fine–Gray model with mixture of continuous and categorical covariant in coinfected patient considering competing risk (death); in Addis Ababa, Ethiopia, (September 2011 to December 2018), n=568.

_t ^m^SHR Std. Err. P>z [95% Conf. Interval]

Hepatitis 0.693309 .0832022 0.002 .5479947 .8771569

sex 1.001302 .1014121 0.990 .8210226 1.221166

age 1.148288 .0869272 0.068 .9899502 1.33195

education 1.070553 .0527625 0.167 .9719785 1.179126

Marital status .9569909 .0536994 0.433 .8573231 1.068246

Baseline cd4 1.339888 .0855247 0.000 1.182324 1.51845

^m^Sub-distribution hazard ratios:
