# Supplementary material for: Retention on antiretroviral therapy in person with HIV and viral hepatitis coinfection in Ethiopia: a retrospective cohort study
Source: BMC Public Health. 2022 Apr 4;22:644. doi: 10.1186/s12889-022-13025-y (PMC8978407; doi:10.1186/s12889-022-13025-y)
Supplement: Supplementary file 5 — Additional file 5. [file 12889_2022_13025_MOESM5_ESM.docx]

**Supplement Table 5a. Sensitivity analysis (excluding TB cases):** Adjusted and unadjusted hazard ratios of retention in coinfected patients with the use of the parametric Gompertz regression model, in Addis Ababa, Ethiopia; (September 2011 to December 2018)

| **Outcome** | Crude Hazard Ratio | | | Adjusted Hazard Ratio^n^ | | |
| --- | --- | --- | --- | --- | --- | --- |
|  | Haz. Ratio  [95% CI] | Standard  Error | *p-value* | Haz. Ratio  [95% CI] | Standard Error | *p-value* |
| **Retention** | | | | | | |
| at 12 months | 0.79(0.62-0.99) | 0.922 | 0.044 | 0.75(0.59-0.96) | 0.094 | 0.026 |
| **Attrition (death and LTFU)** | | | | | | |
| Overall attrition | 1.65(1.05-2.58) | 0.375 | 0.027 | 1.62(1.00-2.64) | 0.403 | 0.050 |

^n^The model was adjusted for age, sex, marital status, education, and baseline CD4 count.

LTFU: Loss-to-follow-up; Overall attrition= LTFU & death over the whole follow-up period

TB: Tuberculosis

**Supplement Table 5b. Sensitivity analysis (excluding HCV cases):** Adjusted hazard ratios of retention in viral hepatitis B HIV positives with the use of the parametric Gompertz regression model, in Addis Ababa, Ethiopia; (September 2011 to December 2018)

**_t | Haz. Ratio Std. Err. z P>|z| [95% Conf. Interval]**

**--------------------------+---------------------------------------------------------------------------------**

HBV | 2.043871 .492854 2.96 0.003 1.274081 3.278764

Sex | 1.189349 .2567988 0.80 0.422 .7789719 1.815921

Age group | 1.100121 .187616 0.56 0.576 .7875453 1.536758

Education | .9880049 .1064572 -0.11 0.911 .7999124 1.220326

M.Status | .8960364 .1087408 -0.90 0.366 .7063597 1.136646

Baseline CD4 | 1.005534 .1614967 0.03 0.973 .7339852 1.377547

WHO stage | 1.892465 .4979823 2.42 0.015 1.129908 3.169659

TB at baseline | .6777763 .1644181 -1.60 0.109 .4213064 1.090372

_cons | .0102415 .006705 -7.00 0.000 .0028385 .0369521

--------------------------+------------------------------------------------------------------------------

/gamma | -.0330122 .0058787 -5.62 0.000 -.0445342 -.0214901

----------------------------------------------------------------------------------------------------------
